# Informal Urban Green-Space: Comparison of Quantity and Characteristics in Brisbane, Australia and Sapporo, Japan

**DOI:** 10.1371/journal.pone.0099784

**Published:** 2014-06-18

**Authors:** Christoph D. D. Rupprecht, Jason A. Byrne

**Affiliations:** 1 Environmental Futures Research Institute, Griffith University, Nathan, Queensland, Australia; 2 Griffith School of Environment, Griffith University, Griffith University, Queensland, Australia; Institute of Agronomy, University of Lisbon, Portugal

## Abstract

Informal urban green-space (IGS) such as vacant lots, brownfields and street or railway verges is receiving growing attention from urban scholars. Research has shown IGS can provide recreational space for residents and habitat for flora and fauna, yet we know little about the quantity, spatial distribution, vegetation structure or accessibility of IGS. We also lack a commonly accepted definition of IGS and a method that can be used for its rapid quantitative assessment. This paper advances a definition and typology of IGS that has potential for global application. Based on this definition, IGS land use percentage in central Brisbane, Australia and Sapporo, Japan was systematically surveyed in a 10×10 km grid containing 121 sampling sites of 2,500 m^2^ per city, drawing on data recorded in the field and aerial photography. Spatial distribution, vegetation structure and accessibility of IGS were also analyzed. We found approximately 6.3% of the surveyed urban area in Brisbane and 4.8% in Sapporo consisted of IGS, a non-significant difference. The street verge IGS type (80.4% of all IGS) dominated in Brisbane, while lots (42.2%) and gaps (19.2%) were the two largest IGS types in Sapporo. IGS was widely distributed throughout both survey areas. Vegetation structure showed higher tree cover in Brisbane, but higher herb cover in Sapporo. In both cities over 80% of IGS was accessible or partly accessible. The amount of IGS we found suggests it could play a more important role than previously assumed for residents' recreation and nature experience as well as for fauna and flora, because it substantially increased the amount of potentially available greenspace in addition to parks and conservation greenspace. We argue that IGS has potential for recreation and conservation, but poses some challenges to urban planning. To address these challenges, we propose some directions for future research.

## Introduction

Dunn et al. argue that global conservation efforts depend on the interest people have in nature conservation, an interest formed largely through experiencing nature within the cities that people inhabit[1]. Informal urban greenspace (IGS) such as vacant lots, brownfields and street or railway verges comprise one part of this urban nature. Research has found that IGS can play a role in exposing city dwellers to nature – as recreational space for residents and an alternative to traditional greenspace (e.g. parks and playing fields)[2]–[4], and as habitat for flora and fauna[5]–[7]. But we presently lack knowledge about the estimated total quantity of IGS in our cities – a key issue, because the quantity of space likely has a strong influence on its potential for recreation and conservation. Questions yet to be answered include: what proportion the different types of IGS contribute to the total amount of IGS in a city, and how does IGS quantity differ between cities? We know little about the spatial distribution (within a city or in different geographical settings) of IGS, its vegetation structure, or its potential accessibility, which are again important factors determining its potential for recreation and conservation. Compounding this problem, scholars presently lack a shared or agreed definition of these taken for granted socio-ecological spaces (though we propose such a definition below). Such a definition is necessary to ensure that researchers are talking about the same concept and vital to creating an integrated research agenda. Finally, we lack a reliable, comprehensive rapid assessment method that can be applied in different geographical contexts and is useful for estimating IGS quantity as a first step in urban planning initiatives to ‘green’ cities. We take up these tasks in this paper.

This paper reports the results of a study that asked the following four research questions: (1) how does the land use proportion of total IGS and individual IGS subtypes differ between urban core areas in two cities? (2) how do the characteristics (distribution, vegetation structure, accessibility) of IGS differ between urban core areas in two cities? (3) does distance from the city center influence IGS quantity, and (4) how accurate is the IGS land use proportion survey method employed for estimating potential IGS quantity? This study contributes new knowledge in two ways. Our study has for the first time examined how much land likely consists of a wide variety of IGS types in an urban core. Second, it represents the first comprehensive examination comparing IGS quantity and type within the urban core area of two cities, potentially allowing scholars to examine IGS composition and quantity in other geographical settings.

## Methods and Data Collection

### Informal urban greenspace (IGS) definition and typology

Cities consist of a patchwork of different spaces, from densely built areas to green space such as urban forests or parklands. But besides these exist also more ambiguous, ‘liminal’ vegetated spaces, that Jorgensen and Tylecote refer to as ‘ambivalent landscapes’ [8]. This heterogeneous group of vacant lots, railway verges, utility corridors and waterway embankments is often overgrown with spontaneous vegetation [7], and is managed only to a limited extent (e.g., vegetation removal to protect power lines from overgrowth). They share ambiguities in land tenure, conservation status, maintenance regimes, use, regulation and legitimacy [9], and are best characterized as liminal spaces. Even street verges and suburban lawns can be liminal. While they may have been planted originally, they are oftentimes a mix of intentionally planted and opportunistic species. Their maintenance level is similar to that of backyard gardens, and depends upon many factors, such as feelings of ownership, cultural beliefs, age, and level of neighbor's surveillance [10], [11]. The concept of liminality is derived from several disciplines, but is well-established in the urban geography literature [12], [13]. It refers to a state of ‘betweenness’, intermediacy, or ambiguity of being – the ‘indeterminacy of loose space’, as Franck and Stevens call it [14]. Liminal spaces are ‘at the margins’, characterized by emergence and flux, fluidity and malleability, and are neither segregated nor uncontained[15].

This liminality presents a challenge for quantitatively surveying such spaces, which we aim to address by proposing a provisional, non-exclusive definition and typology of a form of liminal green spaces we term ‘informal urban green space’ (IGS). For the purpose of this study, we have defined IGS as an explicitly socio-ecological entity, rather than a solely biological or cultural object. IGS consists of any urban space with a history of strong anthropogenic disturbance that is covered at least partly with non-remnant, spontaneous vegetation [5]–[7] and has a history of strong anthropogenic disturbance. It is neither formally recognized by governing institutions or property owners as greenspace designated for agriculture, forestry, gardening, recreation (either as parks or gardens) or for environmental protection (the typical purposes of most greenspace). Nor is the vegetation contained therein managed for any of these purposes. Any use for recreation is typically informal and transitional (e.g. unsanctioned verge gardening).

**Figure 1:**
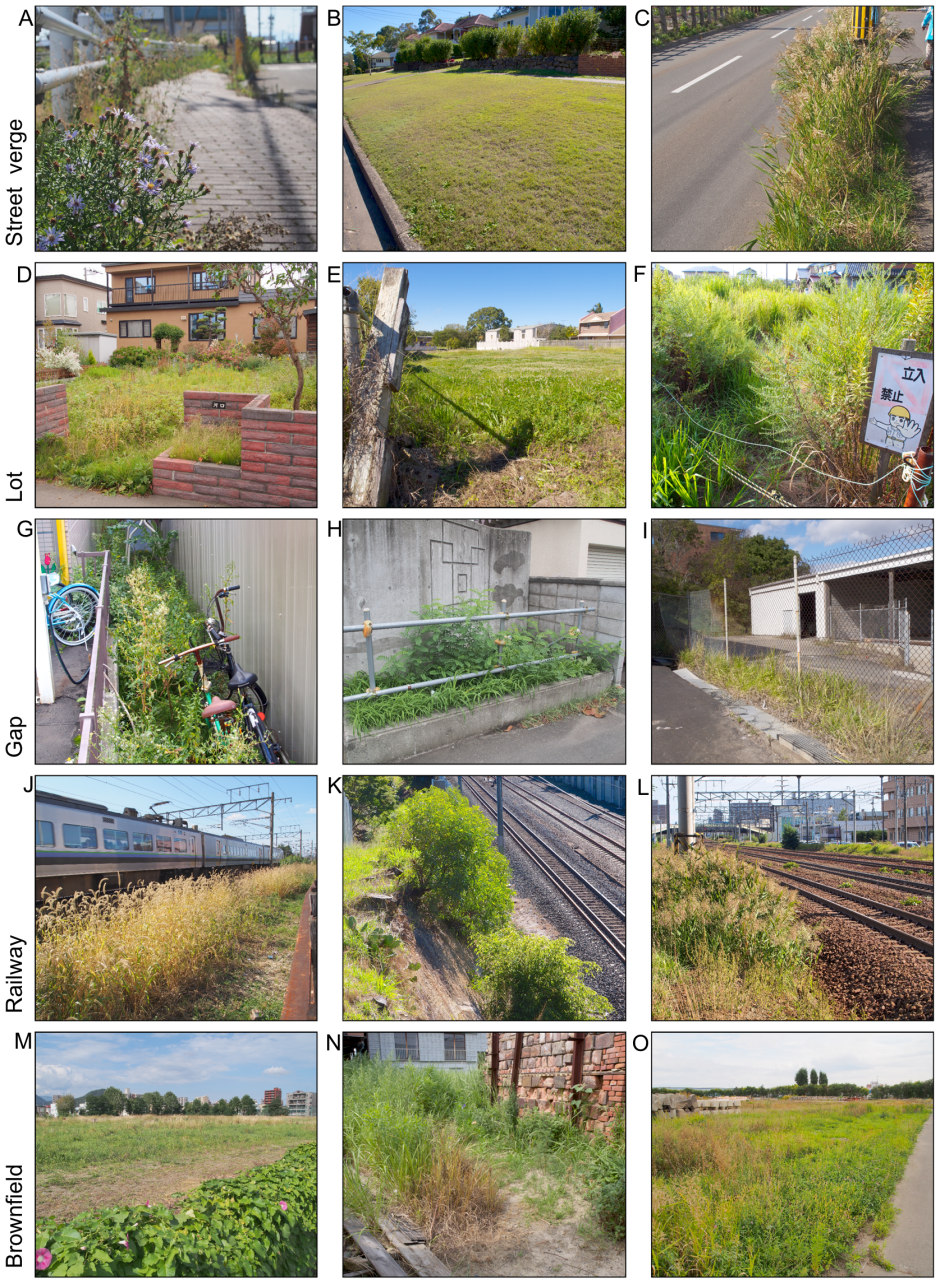
Photos of informal greenspace types following typology in **Table 1**. Street verges: A) Spontaneous herbal vegetation on sidewalk (Sapporo, Japan), B) Unused, highly maintained nature strip with mix of planted and spontaneous vegetation (Brisbane, Australia), C) Spont. herbal vegetation between street and sidewalk (Sapporo). Lots: D) Former residential vacant lot, remains of garden structure still present (Sapporo), E) Long-term vacant lot in residential area (Brisbane), F) Former residential, long-term vacant lot, “no trespassing” sign (Nagoya, Japan). Gap: G) Space with spontaneous herbal vegetation between two buildings, informal storage use (Sapporo), H) Gap with rudimentarily blocked access in front of building (Sapporo), I) Vegetated gap in sealed surface around fence in industrial zone (Brisbane). Railway: J) Annual grass in verge between rail track and street (Sapporo), K) Vegetated cliff next to rail track (Brisbane), L) Vegetated verge and inter-track space (Sapporo). Brownfield: M) Publicly-owned, large vacant tract with grassland and single trees (Sapporo), N) Old city quarter, overgrown former ceramics factory lot (Tokoname, Japan), O) Vegetated area on municipal land for disaster preparation material storage in urban fringe (Sapporo).

**Figure 2:**
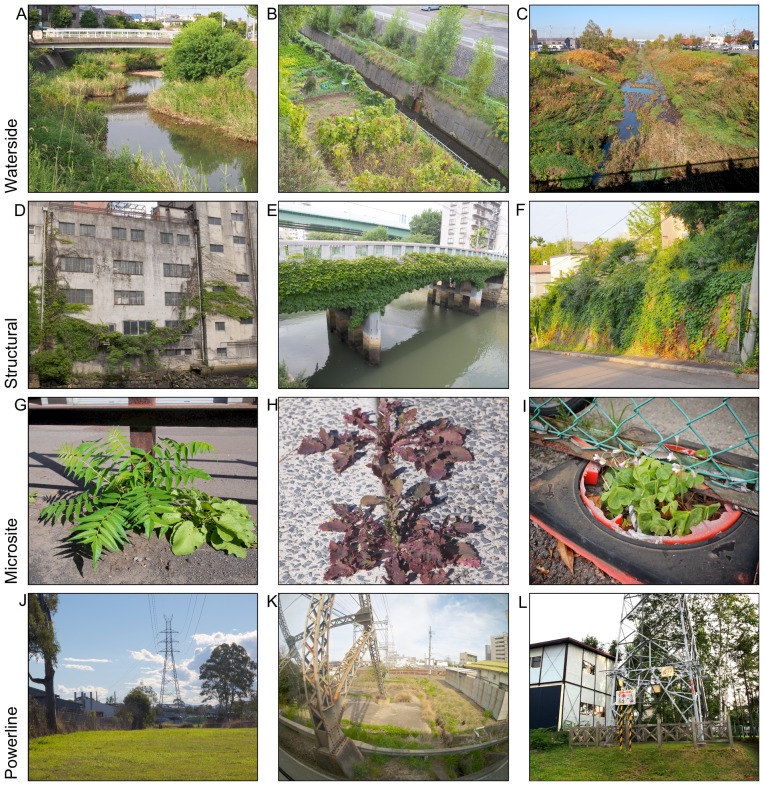
Photos of informal greenspace types following typology in **Table 1** (cont.). Waterside: A) Vegetation on soil deposits in concreted river bed (Nagoya), B) Spontaneous vegetation and informal agricultural use of flood-protection stream banks (Sapporo), C) Spontaneously vegetated anthropogenic river banks (Sapporo). Structural: D) Creeping vines on industrial building (Nagoya), E) Overgrown bridge (Nagoya), F) Concrete soil retention wall completely covered in ivy (Sapporo). Microsite: G) Vegetated crack in asphalt on parking lot (Sapporo), H) Vegetation between two sidewalk plates (Brisbane), I) Plant growing out of degraded traffic cone remains (Nagoya). Powerline: J) Powerline reserve in industrial zone (Brisbane), K) Vegetated area around powerline pylon (near Osaka, Japan), L) Vegetated area around powerline pylon (Sapporo).

IGSs differ in their management (e.g. access, vegetation removal, stewardship), land use and site history, their scale and shape, soil characteristics and local urban context. For example, a small brownfield may be similar to a vacant lot in appearance and size, but their different land use history, vegetation removal periods, and urban context distinguish them. We identified nine potentially different subtypes of IGS: street verge, lot, gap, railway, brownfield, waterside, structural, microsite and power line IGS (Table 1, Figure 1, Figure 2). The subtypes are not exclusive, thus an IGS area may be categorized as multiple subtypes (e.g. street verge and gap). Because this typology recognizes the variety of non-traditional greenspace, it provides a better basis to analyze the implications of IGS for planning and conservation than broad terms such as “wasteland” or “derelict land”. The distinction between IGS and formal greenspace is not binary, but rather characterized by a gradient of informality: formal recognition as recreational space by the owner provides a criterion to identify a local-government owned vacant lot covered with mowed lawn as IGS, but a low maintenance “wild” private garden as formal greenspace. Secondary-growth urban forests (rather than e.g. small patches of woody vegetation on a brownfield) represent a borderline case, but in most cases such forests are recognized for silvicultural or recreational value and thus excluded from the definition of IGS used in this article. For the IGS area survey we only recorded IGS larger than one square meter and therefore also excluded microsite IGS.

**Table 1:** Informal urban greenspace typology.

IGS	Examples	Description	Management	Form	Substrates
**Street verges**	Roadside verges, roundabouts, tree rings, informal trails and footpaths	Vegetated area within 5 m from street not in another IGS category; mostly maintained to prevent high and dense vegetation growth other than street trees; public access unrestricted, use restricted.	Regular vegetation removal (> = once per month); governmental and private stewardship	Small: <100 m^2^, linear	Soil, gravel, stone, concrete, asphalt
**Lots**	Vacant lots, abandoned lots	Vegetated lot presently not used for residential or commercial purposes; if maintained, usually vegetation removed to ground cover; public access and use restricted.	Irregular veg. removal, medium to long removal intervals; private stewardship	Small-medium: <1 ha, block	Soil, gravel, bricks
**Gap**	Gap between walls or fences	Vegetated area between two walls, fences or at their base; maintenance can be absent or intense; public access and use often restricted.	Irregular veg. removal; variable removal intervals; private stewardship	Small: <100 m^2^, linear	Soil, gravel
**Railway**	Rail tracks, verges, stations	Vegetated area within 10 m adjacent to railway tracks not in another IGS category; usually herbicide maintenance to prevent vegetation encroachment on tracks; public access and use mostly restricted.	Regular veg. removal (monthly to yearly); corporate or governmental stewardship	Medium-large: >1 ha, linear	Soil, gravel, stone
**Brownfields**	Landfill, post-use factory grounds, industrial park	Vegetated area presently not used for industrial or commercial purposes; usually no or very infrequent vegetation removal and maintenance; public access and use mostly restricted.	Irregular veg. removal, long removal intervals; corporate and governmental stewardship	Medium-large: >1 ha, block	Soil, gravel, concrete, asphalt
**Waterside**	Rivers, canals, water reservoir edges	Vegetated area within 10 m of water body not in another IGS category; occasional removal of vegetation to maintain flood protection and structural integrity; public access and use often possible with some restrictions.	Irregular veg. removal, long removal intervals; governmental stewardship	Small-large: >10 m^2^ to >1 ha, linear	Soil, stone, concrete, bricks
**Structural**	Walls, fences, roofs, buildings	Overgrown human artifacts; often vertical; occasional removal of vegetation to maintain structural integrity; public access and use mostly restricted.	Irregular veg. removal, medium to long removal intervals; varying stewardship	Small: <100 m^2^, block	Soil, stone, gravel, wood, metal
**Microsite**	Vegetation in cracks or holes	Vegetation assemblages in cracks, may develop into structural IGS; maintenance can be absent or intense	Irregular veg. removal, variable removal intervals; variable stewardship	Very small: <1 m^2^, point	Deposits, soil, stone, concrete
**Power line**	Power line rights of way	Vegetated corridor under and within 25 m of power lines not in another IGS category; vegetation removed periodically to prevent high growth; public access and use mostly unrestricted.	Regular veg. removal (less than yearly); utility or governmental stewardship	Medium-large: >1 ha, linear	Soil

### Study locations

**Figure 3:**
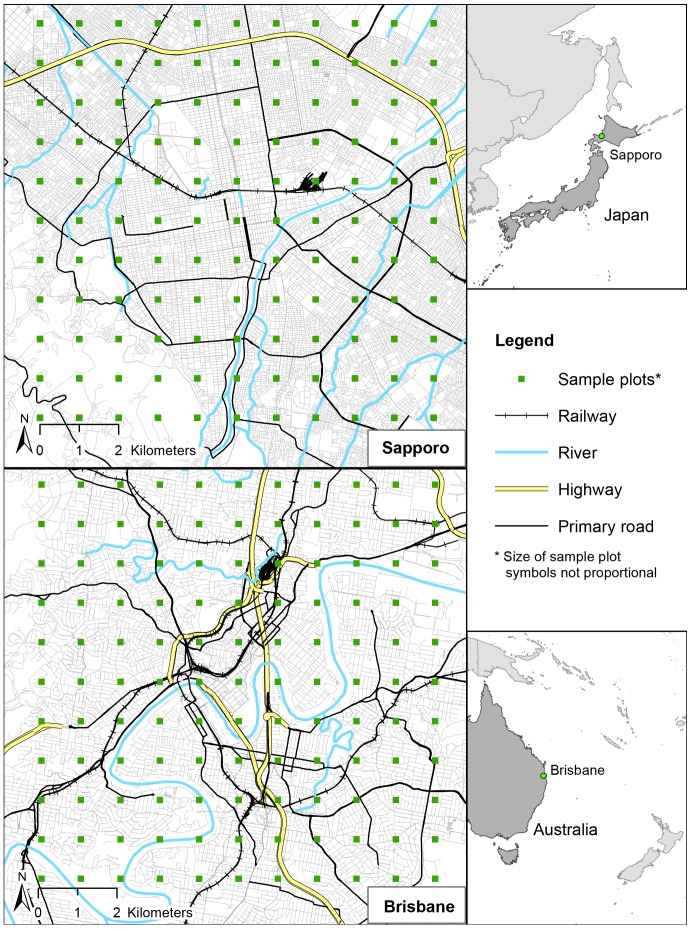
Study locations including sampling sites: Brisbane, Australia (left) and Sapporo, Japan (right).

Brisbane (Queensland, Australia) and Sapporo (Hokkaidō, Japan) were chosen as case study cities, because research that examines IGS outside of Europe and the USA is relatively scarce. The two case study cities have similarities and differences that lend them well to comparison (Table 2); they thus provide excellent opportunities for a cross-cultural research. Both cities are relatively young (being founded in the 19^th^ century) and they saw most of their growth during the 20^th^ century, especially in the post-second world-war period. Their close geographical size is complemented by a similar urban morphology. Both cities are built around a dense central business district, are situated near to the coast and upland regions, and are intersected by a central river (Figure 3). These similarities contrast with differences in population density, population growth forecasts, and available parks and other greenspaces.

**Table 2:** Comparison of cities containing the survey areas.

Characteristics	City of Brisbane (LGA)	Sapporo
Founded	1824, city status 1902	1868, city status 1922
Population	1,089,743 (2011) (2031: 1,27 million)	1,936,189 (2013) (2030: 1,87 million)
Area	1,338 km^2^	1,121.12 km^2^
Pop. density	814/km^2^	1,699/km^2^
Peak density	>5,000/km^2^	>8,000/km^2^
Climate	Humid subtropical (Cfa)	Humid continental (Dfa)
Industry	Tourism, resources, retail, financial services, agriculture hub, education	Tourism, retail, IT, agriculture hub, resources, education
Greenspace	Local parks: 3,290 ha (32 m^2^/capita)	Parks: 2,345 ha (12.3 m^2^/capita)
	All parks: 11840 ha (115 m^2^/capita)	All greenspace: 5,508 ha (28.9 m^2^/capita)
Park area planned	40 m^2^/capita, minimum 20 m^2^/capita	“No greenspace loss, park renovation”

Sources: [46]–[51].

While Sapporo has seen rapid growth throughout the second half of the 20^th^ century and now has a population of about 1.9 million, its population is now stagnating and is predicted to decline in the future. In contrast, Brisbane has a population of around 1 million but is still growing quickly (Table 2). This difference in population development is of particular interest as both expanding cities [16] and shrinking cities [17] have important impacts on urban greenspace provision.

In both cities, formal greenspace consists of networks of over 2,000 public parks, most of them small local parks. Brisbane has 3,290 ha of local parkland (32 m^2^/capita), whereas Sapporo has 2,345 ha (12.3 m^2^/capita) (Table 2). All parks in Brisbane form an area of 11,840 ha (115 m^2^/capita), while all greenspace in Sapporo forms an area of 5,508 ha (28.9 m^2^/capita). These areas include forested hillsides in the southwest of both cities, providing recreational benefits to residents and habitat to wildlife.

### Research design

**Figure 4:**
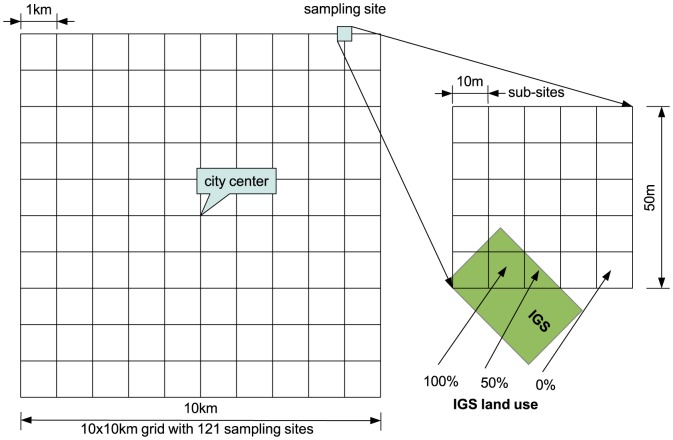
Research design: sampling sites on gridline intersections, with sub-sites and example of IGS percentage calculation.

**Figure 5:**
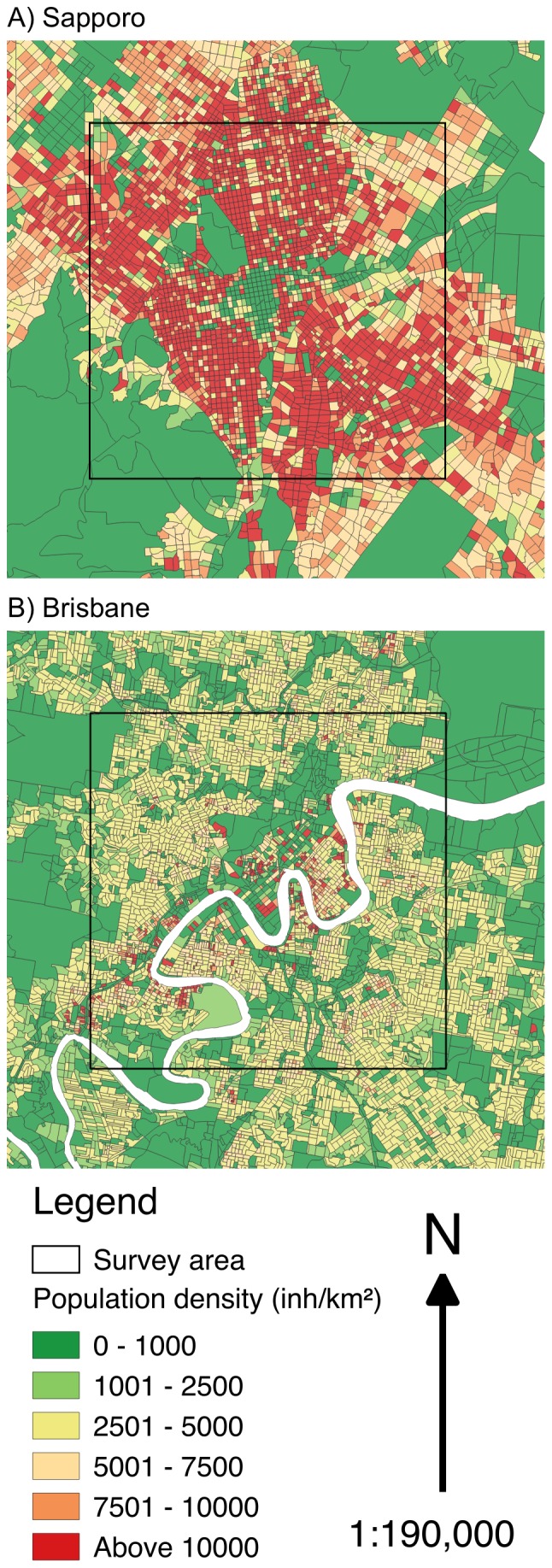
Survey areas and population density of study locations: A) Sapporo, B) Brisbane.

To be able to measure the proportion of land use consisting of IGS and compare it between the survey areas in Brisbane and Sapporo, we used a systematic grid sampling design[18], [19]. We placed 121 sampling sites of 50 m by 50 m each on the intersecting lines of a 10 km by 10 km grid, centered on the city centers (Figure 4, File S1 Sapporo sampling sites, File S2 Brisbane sampling sites). Surveying only the central area of a city rather than the whole allowed us to assess a large area despite limited resources, while still covering most of the densely populated areas where access to greenspace may be difficult[16] (Figure 5). This kind of rapid assessment technique can provide an efficient estimate of land use proportions, and can later be followed up with a more detailed, finer resolution assessment if necessary. The General Post Office (Brisbane) and Sapporo City Office (city hall) were chosen as city centers, following common practice in Australia[20], [21] and Japan[22], [23]. There is no internationally accepted method for determining a city center. There was a one-kilometer distance between any two adjacent sampling sites. Each 2,500 m^2^ sampling site was divided into 25 sub-sites of 10 m by 10 m for a total of 3,025 sub-sites to facilitate land use assessment (total surveyed area 302,500 m^2^ or 0.299% of the square enclosing all sampling sites (101,002,500 m^2^)).

### Land use assessment

We used a three-step process to measure the percentage of IGS and other land uses. First, we created a geographic information system (GIS) layer with site locations and projected it on publicly available high-resolution aerial photography data (Google Earth in Brisbane, see http://www.google.com/earth/; Microsoft Bing Maps in Sapporo, see http://www.bing.com/maps/). Second, we surveyed land use type in the field for each sub-site and recorded land use percentage for small land use areas assessable on the ground. This was conducted using a measuring tape and visual estimation for inaccessible site parts (physically or marked with entry-forbidden signs). Sub-sites (25 sub-sites 10×10 m each) for all 121 sampling sites per city were created, as smaller sites allow both easier tape measurement and easier visual estimation. Only land use of one square meter or more was recorded, smaller areas were included in adjacent land use. Land use types, changes in land use since production of the aerial photography, building and land use borders were added to printed field data entry sheets containing GIS-layer and aerial photography. Land use was categorized using the IGS typology (Table 1, except microsite IGS) and a customized land use category system (Table S1. Land use category system). This was produced loosely based on the Brisbane land use code system[24], [25] and adapted to suit the project after pilot tests. It was further amended in the field if land uses were encountered that could not be properly recorded with the existing categories (e.g. mixed multi-story land use such as bridges or commercial/residential mixed buildings). Extremely rare land uses were filed under the sub-category name “Other” in the category “Other” (category nomenclature followed a category/sub-category system, e.g. “Private greenspace - garden”), recorded and described in a comment field. We documented the site and its surroundings with photographs. Location data was recorded using a handheld GPS device (Trimble Juno ST) at an accessible part of the site edge or at up to 20 m from a site edge. No permission to carry out this study was necessary, as the survey method was designed to work without direct site access, and was conducted on publicly accessible land only. Sites or parts of sites located on private, military or access-restricted conservation land were surveyed visually from publicly accessibly land if possible (likewise for vegetation structure and accessibility), or surveyed via aerial photography only (see above). Data collection for this paper did not involve endangered or protected species.

For the final step, we individually estimated percentages on paper in each 10×10 m sub-site for each land use category present in the sub-site. One percent of land use in each of those sub-sites equals one square meter. For complex sites, additional support lines were drawn across the aerial photo, dividing each sub-site into four 5×5 m sites that each represented 25%. Where necessary, these were further divided into 12.5% or 6.25% blocks. To improve the quality of percentage estimates we used non-GIS-compatible high-resolution aerial photography by NearMap (Brisbane, see https://www.nearmap.com/), the photographic collection produced in the field, and Google Street View (for orientation purposes, see https://www.google.com/maps/views/streetview). In one case a sampling site had to be revisited to re-assess a present IGS. Automating this percentage calculation using software was considered but deemed not feasible for the limited number of sampling sites, as the variable quality and nature of the aerial photography (e.g. perspective distortions of higher buildings) used would have required sophisticated software and labor-intensive checks. Future studies of a larger number of sampling sites or cities should, however, consider the use of such software tools.

### Vegetation structure assessment

**Figure 6:**
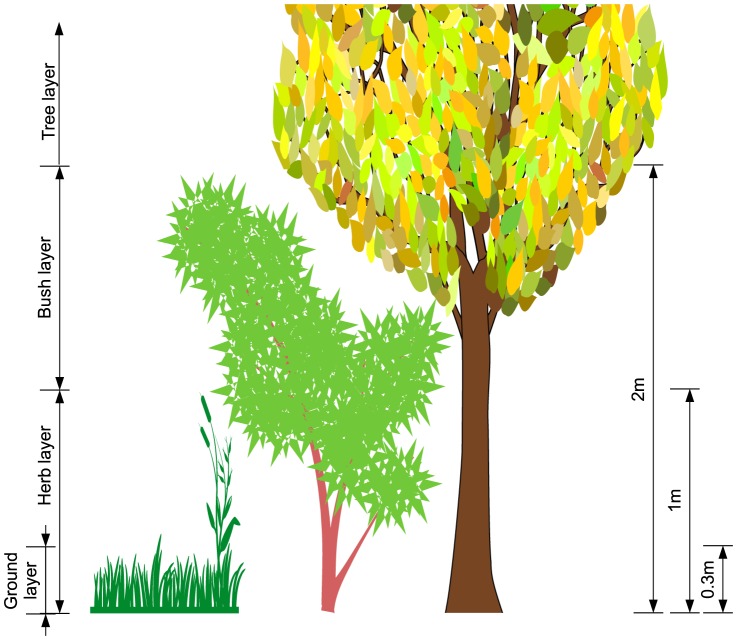
Vegetation structure assessment: tree layer, bush layer, herb layer and ground layer heights.

For all IGS types, we visually estimated (with the help of measuring tape) vegetation cover percentage of four different vegetation strata (Figure 6): 1) tree layer cover (all vegetation >2 m height), 2) bush layer cover (all vegetation between 1 m–2 m height), 3) herb layer cover (all vegetation under 1 m, if vegetation between 30 cm and 1 m height is present), and 4) ground cover (all vegetation under 30 cm, if vegetation between 30 cm and 1 m is not present). Tree layer and herb layer cover are independent, while herb and ground cover are mutually exclusive and thus cannot exceed 100% combined coverage in one IGS. While IGS was defined as vegetated space, ground cover percentage does not have to reach 100% if ground vegetation cover is patchy and includes bare ground (e.g. 10 m^2^ IGS area covered to 50% by herbal layer vegetation, 30% ground layer vegetation, and 20% patchy 1∶1 ground layer vegetation/bare ground mix was recorded as 50% herb cover, 40% ground cover). Vegetation cover height of (possibly vertical) structural IGS was measured in a 90° angle from the substrate.

### Accessibility assessment

**Figure 7:**
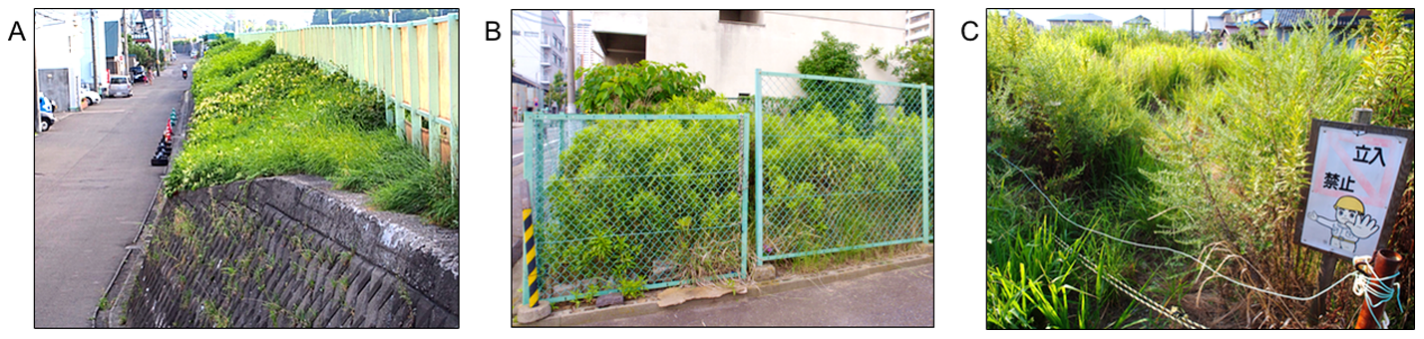
Barriers to IGS access. Example photographs: a) IGS inaccessible due to height and missing ladder; b) IGS completely fenced off; c) IGS access restricted by physical (wire) and symbolic barriers (sign).

We assessed how accessible IGS areas were on a three-level scale derived from prior research into vacant lot accessibility[26], based on the amount of physical or psychological effort necessary to overcome access barriers. As access barriers we included physical barriers such as fences, walls, chains or barbed wire, as well as symbolic barriers such as signs (e.g. “private ground”, “entry forbidden”, “no child play”, Figure 7). IGS were classified as: accessible, if there were no barriers to access, or very low barriers that required only minimal effort to overcome; partially accessible, if a low fence, a “no entry” sign was present, or space was restricted but not too narrow or high to enter and thus required some effort to overcome the barriers; and not accessible, if a high fence, sign warning of injury or other barriers were present that required considerable effort to overcome them.

### Data analysis and statistical methods

We used SPSS (v. 21 and 22, OS X) and R (v. 3.02, OS X) to perform descriptive and inferential statistical analyses. Frequency tables were used to describe quantity of IGS, quantity of IGS types, and IGS characteristics (vegetation structure, accessibility). Initial analysis indicated that the sample data was not normally distributed (P-P plots, skewness and kurtosis tests). We therefore used non-parametric tests, namely a Mann-Whitney U test to test for differences in IGS proportion, and a PERMANOVA test with Euclidean distance matrix to test for differences in IGS type proportions between the two survey areas (using R function *adonis*, see http://cc.oulu.fi/~jarioksa/softhelp/vegan/html/adonis.html). All statistical tests were performed on sampling site scale (N = 121 per survey area) with data aggregated from the sub-sites. To test whether distance of the sampling site from the city center were linked with IGS quantity or IGS type quantity, we used a Pearson correlation. A p-value of 0.05 or smaller was interpreted as statistically significant.

### Method accuracy assessment

To test how accurate the IGS and land use survey method used in this paper was, we compared the results to land use data from GIS data sets supplied by the local city governments. For this purpose, we first combined the geographic features (e.g. polygons representing residential or green space land use) in the city-supplied data sets (ArcGIS 10, UNION), then removed all features outside the smallest possible square containing all sampling sites (ArcGIS 10, CLIP) to calculate the total land use of the features we wanted to compare. In Brisbane, we compared total combined land use percentages of parks (FGSPK), conservation areas (FGSCN), and sports and recreation areas (FGSSR) from our survey with the total greenspace land use percentage from two Brisbane council greenspace data sets (see Table S1 for land use categories). Additionally, we compared total combined residential (all RES categories), garden (PGSGD) and shared greenspace (PGSSG) land use percentages from our survey with the total residential land use percentage of a Brisbane council general land use data set. In Sapporo, we compared park (FGSPK) and sports and recreation area (FGSSR) percentages from our survey with the total non-conservation greenspace land use percentage from a Sapporo City greenspace land use data set. Conservation greenspace was excluded from the comparison in Sapporo because its definition and included greenspace differed substantially between our land use survey and the supplied data set. We then calculated how much the percentages of land use deviate between our land use survey and the supplied GIS data sets.

To check for an accumulation curve and observe the change in land use percentage as sample size increased, we plotted the land use percentage over the number of sites surveyed. Additionally, we plotted the deviation of our land use percentage results from city-supplied datasets (formal greenspace and residential land use in Brisbane, formal greenspace in Sapporo) against the number of sites surveyed. This allowed us to observe what sample size is necessary to achieve a certain level of deviation from the city-supplied datasets.

## Results

**Figure 8:**
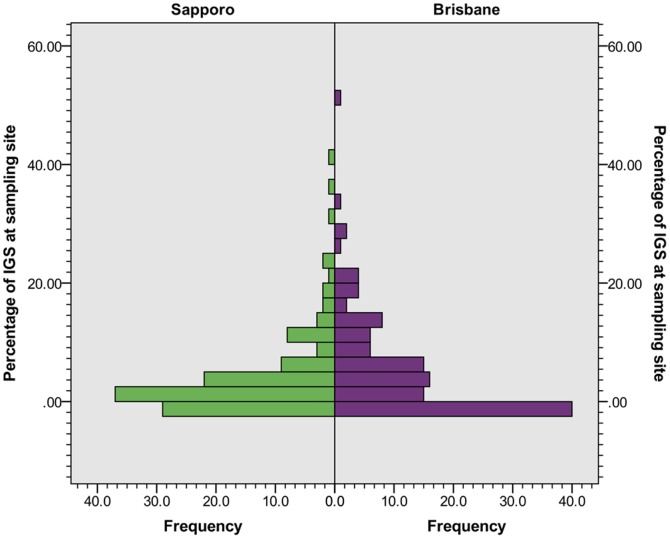
Frequency distribution of IGS land use percentage in sampling sites.

The surveyed area in Brisbane consisted of 6.3% (19,027 m^2^) IGS (Table 3), while the surveyed area in Sapporo consisted of IGS to 4.8% (14,559 m^2^) (Table 4). This difference in IGS proportion was not significant when comparing between the survey areas on site-level (p = .495, N = 242 (121 per survey area), U = 6953.0, z = −.683; for distribution see Figure 8). In the Brisbane survey area, the mean IGS area per site was 157.25 (Mdn = 95, SD = 214.17). In the Sapporo survey area, the mean was 120.32 (Mdn = 41, SD = 186.44). Street verges made up over 80% of IGS in the Brisbane survey area (Table 3), while lots (42.2%) and gaps (19.2%) were the two largest IGS types in the Sapporo survey area (Table 4). In the Sapporo survey area, IGS consisted of more different IGS types and the proportion of individual IGS types showed IGS was more diverse than in the Brisbane survey area (Table 3, 4).The distribution of IGS types between the two survey areas was significantly different (p = .0001, df = 1, Pseudo-F = 19.121, MS = 825762, based on 9999 permutations). In comparison to formal greenspace (parks, sports and recreation, conservation greenspace, and planted verges such as flower-beds) and private greenspace (gardens, shared greenspace, community land, and commercial and industrial greenspace), the area surveyed in Brisbane consisted of more than half as much IGS (6.3%) as formal greenspace (11.6%) and more than one fifth as much IGS than private greenspace (27.4%)(Table 5). In Sapporo, IGS area (4.8%) was almost a third of formal greenspace area (15.4%) and private greenspace (15.0%)(Table 5). Most common non-IGS land use types were small streets (13.3%, INFSS), conservation greenspace (10.7%, FGSCN), and car parks (10.1%, INFCP) in Sapporo, and private gardens (20.7%, PGSGD), small streets (13.6%, INFSS) and residential land use (12.4%, RESLD) in Brisbane (Table S2. Non-IGS land use in Sapporo and Brisbane).

**Table 3:** Quantity of IGS and IGS subtypes in Brisbane survey area.

IGS Type	N*	Quantity (m^2^)	Mean size (m^2^)	Proportion/area (%)	Proportion/IGS (%)
Lot	32	1,433	44.78	0.47	7.53
Gap	22	117	5.32	0.04	0.61
Street verge	643	15,300	23.79	5.06	80.41
Brownfield	15	967	64.47	0.32	5.08
Waterside	7	125	17.86	0.04	0.66
Waterside/verge	–	–	–	–	–
Structural	38	126	3.32	0.04	0.66
Street verge/gap	–	–	–	–	–
Railway	28	959	34.25	0.32	5.04
Lot/street verge	–	–	–	–	–
Powerline	–	–	–	–	–
Total	785	19,027		6.29	
Extrapolated**		6,353,057		6.29	

*N =  number of IGS as recorded in all 3,025 sub-sites.

**Extrapolated to reflect the area of the smallest possible square containing all sampling sites (total square area 101,002,500 m^2^).

**Table 4:** Quantity of IGS and IGS subtypes in Sapporo survey area.

IGS Type	N*	Quantity (m^2^)	Mean size (m^2^)	Proportion/area (%)	Proportion/IGS (%)
Lot	159	6144	38.64	2.03	42.20
Gap	386	2796	7.24	0.92	19.20
Street verge	284	2351	8.28	0.78	16.15
Brownfield	22	1458	66.27	0.48	10.01
Waterside	27	1417	52.48	0.47	9.73
Waterside/verge	5	179	35.80	0.06	1.23
Structural	30	93	3.10	0.03	0.64
Street verge/gap	16	68	4.25	0.02	0.47
Railway	7	43	6.14	0.01	0.30
Lot/street verge	1	7	7.00	0.00	0.05
Powerline	2	3	1.50	0.00	0.02
Total	939	14559		4.81	
Extrapolated*		4858220		4.81	

*N =  number of IGS as recorded in all 3,025 sub-sites.

*Extrapolated to reflect the area of the smallest possible square containing all sampling sites (total square area 101,002,500 m^2^).

**Table 5:** Comparison of IGS, formal and private greenspace in survey areas.

City	Brisbane survey area		Sapporo survey area	
Greenspace type	Area (m^2^)	Area (%)	Area (m^2^)	Area (%)
**Informal greenspace**	19027	6.29	14559	4.81
Parks	16146	5.34	9493	3.14
Sports and recreation	10164	3.36	4423	1.46
Conservation	7641	2.53	32208	10.65
Planted verges	1085	0.36	441	0.15
**Total formal GS**	35036	11.58	46565	15.39
Gardens	62599	20.69	26193	8.66
Shared greenspace	8434	2.79	5052	1.67
Community land	11592	3.83	13210	4.37
Commercial and industrial	387	0.13	776	0.26
**Total private GS**	83010	27.44	45231	14.95
**Total city greenspace**	137073	45.31	106355	35.16

**Figure 9:**
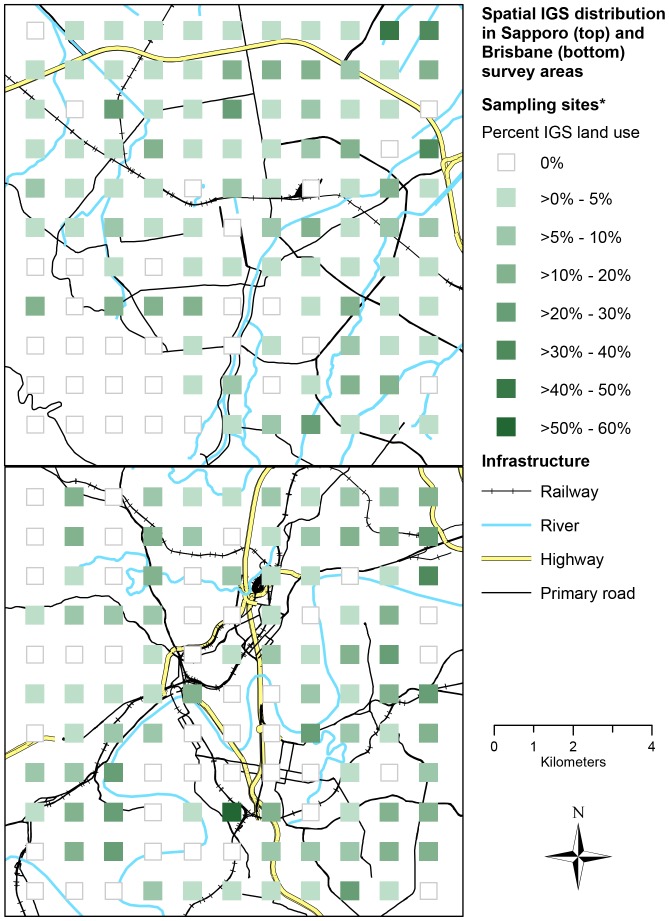
Spatial IGS distribution: percentage of IGS per sampling site in Sapporo (top) and Brisbane (bottom).

We found IGS was present in most of the sampling sites in both cities (Figure 9), with obvious exceptions of sites located in areas with other large-scale land use types (e.g. Brisbane river, Mt. Moiwa in the South-West of Sapporo). For vegetation structure, Brisbane IGS had 27.8% mean tree cover, 7.9% mean bush cover, 21.3% mean herb cover, and 72.8% mean ground cover (Table 6). Sapporo IGS had less mean tree cover (6.5%), similar mean bush cover (7.8%), higher herb cover (43.0%) and subsequently lower ground cover (46.0%) (Table 6). For accessibility in Brisbane, 78% of IGS area was accessible, 7% partially accessible and 15% not accessible (Table 7). In Sapporo, the accessible IGS area (68%) and not accessible area (10%) was smaller, compensated by a larger partially accessible IGS area (21%) (Table 6). Lot, gap and brownfield IGS was more accessible in Sapporo, but street verge and waterside IGS in Brisbane (Table 6). When testing whether the total amount of IGS or the amount of an individual IGS type was correlated with distance to the city center, we found no significant correlations between either total IGS or individual IGS type and distance in either surveyed area.

**Table 6:** Comparison of IGS vegetation structure in survey areas.

City	Brisbane survey area						Sapporo survey area					
IGS Type	N*	Tree (%)	Bush (%)	Herb (%)	Ground (%)	HG (%)**	N*	Tree (%)	Bush (%)	Herb (%)	Ground (%)	HG (%)**
Brownfield	15	0.0	0.0	51.3	34.0	85.3	22	0.0	95.0	100.0	0.0	100.0
Gap	22	0.0	2.3	57.0	21.6	78.6	386	3.2	6.2	45.3	44.4	89.7
Lot	32	23.6	12.5	79.4	11.7	91.1	159	7.6	8.3	36.1	37.7	73.8
Lot/street verge		–	–	–	–	–	1	0.0	0.0	100.0	0.0	100.0
Powerline		–	–	–	–	–	2	0.0	0.0	0.0	100.0	100.0
Railway	28	1.8	6.6	76.8	4.1	80.9	7	0.0	0.0	75.7	12.9	88.6
Street verge	643	31.7	7.8	10.2	85.3	95.5	284	11.7	2.9	34.4	58.9	93.3
Street verge/gap		–	–	–	–	–	16	0.0	0.6	49.4	43.1	92.5
Structural	38	10.5	9.7	73.2	21.3	94.5	30	0.0	6.2	26.3	70.7	97.0
Waterside	7	35.7	21.4	92.9	0.0	92.9	27	0.7	11.9	93.0	7.0	100.0
Waterside/verge		–	–	–	–	–	5	64.0	46.0	100.0	0.0	100.0
**Total IGS**	**785**	**27.8**	**7.9**	**21.3**	**72.8**	**94.0**	**939**	**6.5**	**7.8**	**43.0**	**46.0**	**89.0**

*N =  number of IGS as recorded in all 3,025 sub-sites.

**HG  =  combined percentage of herb and ground cover. Herb and ground cover strata add up to 100% minus ground not covered by vegetation.

**Table 7:** Comparison of IGS accessibility in survey areas.

Survey area	Accessibility	Lot	Gap	Street verge	Brownfield	Waterside	WS/SV*	Structural	SV/GP*	Railway	LT/SV*	Powerline	Total IGS
**Brisbane**	Total (N)	32	22	643	15	7	0	38	0	28	0	0	**785**
	Total (m^2^)	1433	117	15300	967	125	0	126	0	959	0	0	**19027**
	Yes (N)	7	3	622	0	7	–	16	–	0	–	–	**655**
	Yes (N%)	22	14	97	0	100	–	42	–	0	–	–	**83**
	Yes (m^2^)	231	10	14433	0	125	–	50	–	0	–	–	**14849**
	Yes (% of area)	16	9	94	0	100	–	40	–	0	–	–	**78**
	Partial (N)	12	6	13	0	0	–	8	–	0	–	–	**39**
	Partial (N%)	38	27	2	0	0	–	21	–	0	–	–	**5**
	Partial (m^2^)	661	23	655	0	0	–	28	–	0	–	–	**1367**
	Partial (% of area)	46	20	4	0	0	–	22	–	0	–	–	**7**
	No (N)	13	13	8	15	0	–	14	–	28	–	–	**91**
	No (N%)	41	59	1	100	0	–	37	–	100	–	–	**12**
	No (m^2^)	541	84	212	967	0	–	48	–	959	–	–	**2811**
	No (% of area)	38	72	1	100	0	–	38	–	100	–	–	**15**
**Sapporo**	Total (N)	159	386	284	22	27	5	30	16	7	1	2	**939**
	Total (m^2^)	6144	2796	2351	1458	1417	179	93	68	43	7	3	**14559**
	Yes (N)	131	178	265	11	15	0	19	12	0	1	2	**634**
	Yes (N%)	82	46	93	50	56	0	63	75	0	100	100	**68**
	Yes (m^2^)	5032	1154	1800	761	1007	0	73	50	43	7	3	**9930**
	Yes (% of area)	82	41	77	52	71	0	78	74	100	100	100	**68.2**
	Partial (N)	17	111	15	11	3	5	7	4	0	0	0	**173**
	Partial (%)	11	29	5	50	11	100	23	25	0	0	0	**18**
	Partial (m^2^)	714	924	441	697	130	179	16	18	0	0	0	**3119**
	Partial (% of area)	12	33	19	48	9	100	17	26	0	0	0	**21.4**
	No (N)	11	97	4	0	9	0	4	0	7	0	0	**132**
	No (%)	7	25	1	0	33	0	13	0	100	0	0	**14**
	No (m^2^)	398	718	110	0	280	0	4	0	0	0	0	**1510**
	No (% of area)	6	26	5	0	20	0	4	0	0	0	0	**10.4**

*WS/SV  =  waterside/street verge, SV/GP  =  street verge/gap, LT/SV  =  lot/street verge.

**Figure 10:**
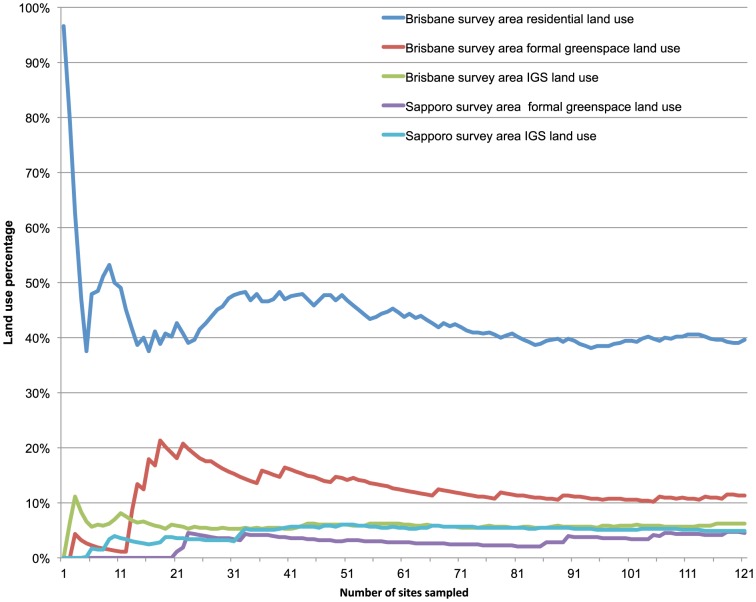
Change in total land use percentage with increasing sample size.

**Figure 11:**
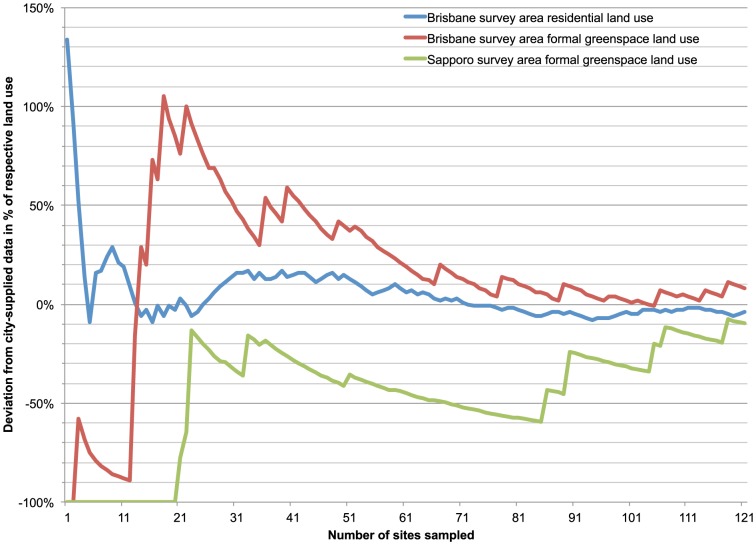
Decrease in deviation of total land use percentage from city-supplied datasets with increasing sample size.

When measuring the accuracy of our land use survey method, we found the combined percentage of parks, conservation and sports and recreation land use in our survey (11.2%) deviated 8.4% from the combined greenspace land use percentage in Brisbane Council datasets (10.4%). The combined percentage of residential, garden and private shared greenspace in our survey (39.5%) deviated -4.2% from the residential land use percentage in the Brisbane Council dataset (41.3%). In Sapporo, the combined percentage of non-conservation greenspace in our survey (4.8%) deviated −6.9% from the non-conservation greenspace percentage in the Sapporo City dataset (5.1%). As a result, the accuracy of our method was over 90% in both cities when comparing land use percentages of around 5% or more with those of official datasets. A visualization of the change in land use percentage with increasing sample size showed that for a common land use type (residential), good accuracy was reached at a sample size of around 70, while for the rare land use types a sample size of around 90 was necessary (Figure 10). A deviation from the city-supplied datasets of less than 10% is reached at 60 sampled sites for the residential land use type, but only at 120 sampled sites for the formal greenspace land use types (Figure 11).

## Discussion and Conclusions

This study has found similar proportions of IGS in both survey areas. While this could indicate other urban areas may contain a similar percentage of IGS, the conclusions we can draw are limited by the sampling design used. The similarity of the study cases (e.g. age and spatial structures of the cities, size and shape of the survey areas; see Methods) may be partly responsible for the similarity in IGS proportions, so results may vary across survey areas with different characteristics. The survey areas we compared differ in their population density (Figure 5) and cultural context. These two factors seemed to have little influence on the proportion of IGS in the survey areas and its accessibility. However, they may explain the differences in IGS types and vegetation structure. For example, higher population density may influence the amount of land dedicated to street verges through planning policy. The rapid growth Brisbane is experiencing may limit the proportion of lot type IGS, as a high demand for land available for development possibly reduces the time land remains vacant before redevelopment. IGS was also widely, but not equally distributed throughout the survey areas (Figure 9). To better understand the factors driving IGS occurrence across different cities, future research should seek to compare IGS in multiple locations. Additionally, possible links between IGS occurrence and factors such as other land use types (e.g. formal green space, industrial, residential) or average income around the site could be explored.

The sampling design used in this study has limitations that make it unsuitable for assessing factors of importance to urban conservation, such as the size of individual IGS or the distribution of IGS sizes. Such information is valuable and has been recorded by prior research into roundabouts[27] and vacant lots[26], but these studies require a different sampling design to studies aiming at measuring the percentage of IGS land use in a survey area. However, for research on the size of individual IGS, linear IGS (e.g. railway verges) and microsites represent challenges as they lack clear boundaries.

Most prior research on IGS has pointed out its potential without knowing what proportion of cities consists of IGS. Having this knowledge allows us to identify some potential policy implications that IGS has for recreational use and conservation, by considering its area compared to other greenspace types. IGS accounts for about 14% of total greenspace in the survey areas (Table 5). This suggests IGS may represent an important source of green space, an aspect further emphasized by the fact that the proportion of IGS present in both survey areas was similar. Furthermore, in both survey areas over 80% of IGS was accessible or partly accessible. However, the limitations of the sampling design discussed above also apply here. Additionally, these findings represent just the first necessary step to understand the potential of IGS for recreation and IGS. Further research is needed to clarify the degree to which IGS is used by residents, whether accessible sites are actually accessed, and which factors may influence residents' perception and appreciation of IGS. These questions are particularly important, because IGS is different from formal greenspace. It lacks common park facilities such as seating or toilets, its informal nature can cause especially adults to perceive it as unsafe or dangerous[28], and the liminal nature of these spaces may limit the degree to which they can be planned – a challenge for urban planners. Yet, being different can also be an advantage. IGS can offer residents an alternative experience to formal greenspace[2], [29], [30], such as opportunities for children to test themselves in a non-controlled environment[31].

The results also have implications for urban conservation. Research has shown IGS plays a role in providing habitat to fauna and flora[32], [33] as well as in connecting habitat in and between cities[34], [35]. The amount of IGS we found (three times that of conservation greenspace in Brisbane and close to 50% in Sapporo survey areas), its distribution throughout the survey areas, and the relatively complex vegetation structure (Table 6) suggest IGS' role for urban conservation may be more important than previously assumed. Sites with IGS completely surrounded by sites without IGS were rare, suggesting a potential connectivity for species present in IGS (Figure 9). The lack of difference in IGS proportion between the survey areas raises the possibility that a similar percentage of IGS may also be available for conservation in other cities, although the limits of the sampling design discussed above need to be taken into account. The differences in vegetation structure and individual IGS types also imply its actual conservation potential likely depends on its local characteristics. There are concerns about the opportunistic type of species IGS tends to favor[36], and the possible function of IGS as a reservoir or corridor for biological invasions[37]. But research has not always confirmed such connectivity for invasive species[35], and many opportunistic species are adapted to their local environment – they represent what could be called the “de facto native vegetation of the city” [7], [38]. As Dunn et al. suggest, the opportunities for nature experience IGS can provide to residents may also be vital for conservation efforts, even beyond urban areas[1].

The potential importance of IGS for urban recreational use and urban conservation has implications for urban and environmental planning. Planners may need to re-think their negative view of ‘vacancy’ in the urban landscape[39] and acknowledge the benefits of residents' informal creativeness[40]. Expectations for parks, conservation greenspace and private greenspace such as gardens as sole providers of recreational and conservation benefits might need to be reevaluated. Reducing barriers to IGS access[26] or reusing IGS as community gardens[41] are ways to improve IGS utility for residents, but planners should refrain from too much intervention. Some scholars assert that IGS does not necessarily need to be tamed, but should be valued for its informality, ambivalence and special aesthetic [14], [29]. In the case of some IGS, freedom of purpose can mean freedom from purpose (e.g. abandoned lots), while other IGS (e.g. utility corridors, railway verges) have purpose but may have to capacity to accommodate additional, informal use. On the other hand, IGS may have negative effects for residents, such as damage to structures caused by spontaneous vegetation [42], [43]. The liminality and legally non-public status of many IGS may also present a challenge to planners, as their influence on IGS characteristics such as accessibility is likely limited. It depends not only on the cooperation of the formal space owners, but issues such as liability for injury involve third parties such as insurance companies[44].

The results of our study emphasize more research on IGS is needed to unlock its full potential. Examining how IGS is influenced by its socio-ecological context would be a valuable starting point for future studies. A quantitative examination of how residents use and perceive IGS could provide into the social aspects. A cross-cultural comparison seems particularly promising, as concepts such as public space may be interpreted differently depending on the cultural context[45]. A possible direction for research on the ecological side could be a comprehensive examination of the value of IGS for biodiversity, either in the form of a systematic literature review providing a synthesis of the many studies focusing on only one or a few IGS types, or in the form of field studies taking into account all IGS types identified in this paper. Finally, replications of the study conducted in this paper in geographic locations around the world would improve our global knowledge of global IGS distribution and provide valuable input for planning policy.

## Supporting Information

Table S1 **Land use category system.**
(XLSX)

Table S2 **Non-IGS land use in Sapporo and Brisbane.**
(XLSX)

File S1 **Sapporo sampling sites.**
(KMZ)

File S2 **Brisbane sampling sites.**
(KMZ)
